# Fine-scale-mapping of *Schistosoma haematobium* infections at the school and community levels and intermediate host snail abundance in the north of Pemba Island: baseline cross-sectional survey findings before the onset of a 3-year intervention study

**DOI:** 10.1186/s13071-022-05404-6

**Published:** 2022-08-16

**Authors:** Lydia Trippler, Said Mohammed Ali, Shaali Makame Ame, Jan Hattendorf, Khamis Rashid Suleiman, Mohammed Nassor Ali, Saleh Juma, Fatma Kabole, Stefanie Knopp

**Affiliations:** 1 Department of Epidemiology and Public Health, Swiss Tropical and Public Health Institute, Allschwil, Switzerland; 2 University of Basel, Basel, Switzerland; 3 Public Health Laboratory—Ivo de Carneri, Wawi Pemba, United Republic of Tanzania; 4 Neglected Diseases Program, Zanzibar Ministry of Health, Mkoroshoni Pemba, United Republic of Tanzania; 5 Neglected Tropical Diseases Program Ministry of Health , Lumumba, Unguja, United Republic of Tanzania

**Keywords:** *Bulinus globosus*, Distance, Elimination, Fine-scale mapping, Freshwater bodies, Interruption of transmission, Mass drug administration, *Schistosoma haematobium*, Surveillance response, Test-and-treat

## Abstract

**Background:**

Schistosomiasis elimination has gained renewed priority in the WHO guidance documents published in 2020 and 2022. The SchistoBreak project, implemented in Pemba, Tanzania between 2020 and 2024, aims to assess new tools and strategies for shifting from elimination as a public health problem towards interruption of transmission. Here we report our baseline findings and discuss implications for future interventions.

**Methods:**

In 2020, human water contact sites (HWCSs) in the study area were geolocated and snail surveys were conducted. A parasitological and questionnaire cross-sectional baseline survey was implemented in 20 communities and their 16 primary schools between November 2020 and February 2021. Urine samples were collected at the school and household levels from individuals aged ≥ 4 years. *Schistosoma haematobium* infection was detected by urine filtration microscopy. Snail, parasitological and questionnaire-derived data were analyzed descriptively, spatially and with generalized estimated equation models.

**Results:**

The intermediate host snail *Bulinus globosus* was detected in 19.8% (33/167) of HWCSs. The overall *S. haematobium* prevalence was 1.2% (26/2196) in school-aged children and 0.8% (31/3893) in community members, with 0.2% (4/2196) and 0.1% (3/3893) heavy-intensity infections, respectively. Children who studied < 1 km away from HWCSs with *B. globosus* had significantly higher odds for a *S. haematobium* infection than those attending a school located > 2 km away (odds ratio [OR]: 5.0; 95% confidence interval [CI]: 2.3–11.1). Individuals living in a house located < 1 km away from HWCSs with *B. globosus* had higher odds than those residing in > 2 km distance (OR: 18.0; 95% CI: 2.9–111.0). Self-reported praziquantel treatment coverage was 83.2% (2015/2423) in schoolchildren in the mass drug administration (MDA) conducted in August 2020. Coverage among adult community members was 59.9% (574/958), but only 34.8% (333/958) took praziquantel correctly.

**Conclusions:**

While the *S. haematobium* prevalence is very low in Pemba, there are many HWCSs with *B. globosus* situated close to schools or houses that pose a considerable risk of recrudescence. To maintain and accelerate the progress towards interruption of transmission, targeted and cost-effective interventions that are accepted by the community are needed; for example, snail control plus focal MDA, or test-and-treat in schools and households near infested waterbodies.

**Graphical Abstract:**

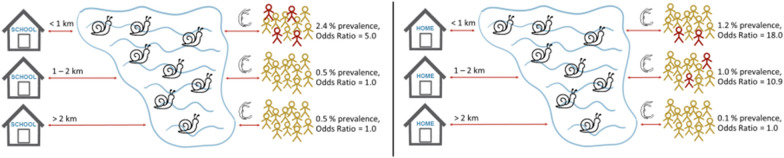

**Supplementary Information:**

The online version contains supplementary material available at 10.1186/s13071-022-05404-6.

## Introduction

Schistosomiasis is a highly debilitating disease endemic in 78 countries worldwide [1]. Its highest burden is concentrated in sub-Saharan Africa, where 91% (42/46) of the countries are affected [2]. Recent model-based estimates indicate, however, that the schistosomiasis prevalence in sub-Saharan Africa has decreased considerably over the past few decades, likely due to the scale-up of preventive chemotherapy programs [3].

With the “Roadmap for neglected tropical diseases 2021–2030” and the “Guideline on control and elimination of human schistosomiasis” published by the WHO in 2020 and 2022, respectively, the global elimination of schistosomiasis as a public health problem by 2030 and the interruption of transmission in selected countries by 2030 gained renewed priority and important guidance [1, 4].

Pemba Island, part of the Zanzibar archipelago in the United Republic of Tanzania, has achieved elimination of urogenital schistosomiasis as a public health problem since 2017 [5–7]. Continued actions are required to sustain and even advance these gains made towards interruption of transmission [4]. The SchistoBreak project, implemented in 20 communities in the north of Pemba Island in 2020 and ongoing until 2024, aims to assess new tools and strategies for exactly this purpose [8]. Largely in accordance with the new WHO recommendations 2, 4 and 5 [4], three strategies are implemented in the smallest administrative areas (shehias) of the study area dependent on prevalence: (i) shehias with a *Schistosoma haematobium* prevalence ≥ 3.0% in the annual school-based survey and/or ≥ 2.0% in the annual community-based survey will receive regular preventive chemotherapy plus snail control and behavior change measures to further reduce transmission and infection prevalence; ii) shehias with a *S. haematobium* prevalence < 3.0% in the annual school-based survey and < 2.0% in the annual community-based survey will receive targeted test-treat-track interventions of at-risk populations plus focal snail control to prevent recrudescence; and (iii) all shehias implement passive surveillance and treatment of cases in their health facilities [8].

The results of the SchistoBreak study will generate important evidence to support and potentially reshape existing guidelines and provide new insights into interrupting *S. haematobium* transmission in Pemba and other low-prevalence settings in the future. Here, we report the baseline findings from 2020/2021 and discuss implications for present and future risk assessments and intervention approaches.

## Methods

### Study setting

The SchistoBreak study is being conducted on Pemba Island, which is part of the Zanzibar archipelago in the Indian Ocean, located around 30 km east of the Tanzanian mainland. Pemba is divided into four main districts, which are further split at the sub-district level into 129 small administrative areas, called shehias [9]. Our study area covers 20 shehias, which in this paper are referred to as “implementation units” (IUs), across the two districts of Micheweni and Wete in the north of Pemba. Urogenital schistosomiasis, caused by *S. haematobium*, used to be highly endemic in Pemba in the 1980s [10, 11]. No other *Schistosoma* species infecting humans are known to be endemic on the Zanzibar islands [12]. Interventions to control morbidity of urogenital schistosomiasis in Pemba started with test-and-treat activities in the 1980s [13] and later expanded to regular island-wide mass drug administration (MDA) campaigns in the early 2000s [14]. More intense elimination efforts were carried out as part of a cluster-randomized trial on islands of the archipelago, Unguja and Pemba, between 2011/2012 and 2017 [5, 7]. The most recent results published from Pemba indicate a low overall *S. haematobium* prevalence of 3.4% among schoolchildren aged 9–12 years and 0.4% among adults aged 20–55 years in 2020 [6]. A considerable temporal and spatial heterogeneity exists, however, with many low-prevalence areas and a few remaining hotspots [5–7].

### Characteristics of IUs included in the survey

To gain a comprehensive picture of the educational and environmental characteristics of the study area, a survey was conducted in the 20 study IUs between February and July 2020, with a 3-month interruption due to COVID-19 that resulted in a lockdown by the Tanzanian government from March to June 2020. Details on the IU characteristics survey have been published elsewhere [8]. In brief, initial interviews were held with the leader (sheha) of each IU who provided information on the respective IU. Data were collected on the population size and number of housing structures, the number and type of schools and the number and locations of natural open freshwater bodies and public clean water sources. Subsequently, each nursery, primary and secondary school and all Madrassas (Islamic schools) in the IUs were visited and data were collected on each school’s geolocation and the number of children enrolled in 2020. Finally, all known human water contact sites (HWCSs) were located in the IUs, their geolocation recorded and data collected on the type and characteristics of the water bodies and the abundance of freshwater snail species—specifically the intermediate-host snail *Bulinus globosus*. Individuals who were present at the HWCSs at the time of the visit were invited to share their opinion on their community’s needs for better protection against *S. haematobium* infections.

Additionally, in September 2021, all health facilities used by individuals living in the IUs were visited, their geolocation recorded and data collected about the type of the health facility (primary health care unit [PHCU] vs hospital, and public vs private). All data were captured with Open Data Kit (ODK) software (www.opendatakit.org) installed on Samsung Galaxy Tab A tablets (Samsung Electronics, Seoul, South Korea).

### Cross-sectional parasitological surveys

To assess the prevalence of *S. haematobium* infections in Pemba at baseline, a cross-sectional parasitological survey was conducted in the 20 study IUs from November 2020 until March 2021. Based on the survey results, the IUs were stratified into hotspot IUs or low-prevalence IUs to assign respective measures for the intervention period following the baseline survey. The survey included a school-based and a community-based component to obtain an accurate picture of *S. haematobium* infections in: (i) the school-aged population, which is considered to be at the highest risk of infection [15], and (ii) the whole population, including all individuals aged ≥ 4 years, in the study areas.

#### School-based survey

The school-based cross-sectional survey was conducted in the largest public primary school of each IU [8]. All children aged ≥ 4 years enrolled in the school were eligible to participate in the study. Each school was visited on 2 consecutive days. On day 1, one class (A, B, C, D…) in each of seven grades (nursery school, and grade 1 to grade 6) was selected based on a computer-generated randomized list. In each selected class, the students were asked to line up, with girls and boys lining up separately, and each third child in the lines was selected until a total of 25 children per class, half girls, half boys, were included in the study. Demographic information, such as names, age and sex, was collected from all selected students. The selected children were also asked about any travel in the previous 6 months and about their participation in the school-based MDA with praziquantel conducted in August 2020. Each selected child received a consent and information form to be signed by their parent or legal guardian and to be returned the following day. Children aged ≥ 12 years were additionally asked to provide their own written assent for participation. On day 2, between 9 AM and 2 PM, a urine collection cup was provided to all children who had returned a signed consent form. Each cup was labeled with a unique identifier code. The children were advised to fill their cup with their own fresh urine sample and subsequently submit it to the field enumerators.

#### Community-based survey

The community-based cross-sectional survey was conducted in each of the 20 study IUs [8]. In preparation for the survey, 70 housing structures per IU were randomly selected from available shape file data provided by the Zanzibar Commission for Lands to the Zanzibar Neglected Diseases Program [16]. Field enumerators identified the geolocation of each selected housing structure with ODK, combined with the offline navigation application “MAPS.ME” (www.maps.me). Details of the randomization process of housing structures and the GPS-based fine-scale mapping approach are described elsewhere [16]. Each IU was visited for 3 consecutive days. On day 1, the enumerators visited all selected housing structures in the IU, explained the aims of the study in Kiswahili to all household members present at the time and invited them, as well as those family members who were absent at the time but who were returning to the residence later in the day, to participate. Once written informed consent was obtained, an interview with one adult household member who was present was conducted. The questionnaire covered sociodemographic details (age, name, sex) of all household members, as well as information on participation of the interviewed person in the last community-based MDA, their opinion on MDA as a measure against schistosomiasis and their travels in the previous 6 months. All household members aged ≥ 4 years were eligible to submit a urine sample for *S. haematobium* testing. For this purpose, an information and consent form was provided to each eligible household member to be signed by themselves if they were ≥ 18 years of age or else by their parent or legal guardian. Children aged ≥ 12 years were additionally asked to provide their own written assent for participation. Moreover, each eligible household member was given a plastic cup for urine collection that was labeled with a unique identifier code and a sticker with a specific drawing (for example of a boat, cat, or cow). The stickers were linked to the same stickers on a paper list, where the names of the household members were indicated in addition to the stickers to allow a clear association of drawing and name and hence to avoid confusion of the urine cups between the household members. All consenting participants were asked to fill the container with one sample of their own urine until the next morning. On day 2, the enumerators revisited the households from day 1 for urine container collection and for collection of signed consent and assent forms. All residential houses where nobody was present on day 1 were revisited. On day 3, all signed consent and assent forms and urine cups that had not been submitted on day 1 or 2 were collected.

#### Laboratory examinations

All urine samples were transferred to the Public Health Laboratory—Ivo de Carneri (PHL-IdC) in Wawi, ChakeChake for analysis on the same day. Each sample was examined for microhematuria using reagent strips (Hemastix; Siemens Healthcare Diagnostics AG; Zurich, Switzerland). Additionally, each sample was filtered through a 13-mm fabric filter (Sefar Ltd., Bury, UK) placed in a Swinnex plastic filter holder (MilliporeMerck KgaA, Darmstadt, Germany) using a 10-ml plastic syringe and examined for the presence and number of *S. haematobium* eggs under a light microscope.

### Data management

All laboratory results for both the school- and community-based surveys were captured on paper and double entered onto electronic spreadsheets (Excel 2010; Microsoft Corp., Redmond, WA, USA). Double entered data were compared and cleaned with R version 4.0.3 (www.rproject.org) and Stata/IC 16.1 (StataCorp LLC, College Station, TX, USA). Discrepant results were verified against data on the corresponding original paper forms and re-entered correctly. All registration and questionnaire data captured in ODK were sent to the ODK central server hosted at the Swiss Tropical and Public Health Institute in Basel, Switzerland. For statistical analyses, coded laboratory results were merged with the registration and/or questionnaire information using R and Stata/IC. Participant names were kept in a separate file and only relinked with the coded results to inform all participants about their infection status with *S. haematobium*.

### Statistical methods

Statistical analyses were performed using R version 4.0.3. The median number of public water taps reported by the sheha was determined per 1000 IU inhabitants and rounded to whole number.

The geolocations of nursery, primary and secondary schools and Madrassas identified in the IU characteristics survey, the number of children enrolled in the schools and all identified HWCSs in the study area together with the number of *B. globosus* found were mapped with ArcMap version 10.6.1 (ESRI, Redlands, CA, USA).

All eligible individuals who provided a urine sample were included in the parasitological analysis. A participant was considered to be *S. haematobium*-positive if ≥ 1 egg was detected by urine filtration. *Schistosoma haematobium* infection was classified into two intensity categories: (i) light intensity (1–49 *S. haematobium* eggs/10 ml), and (2) and heavy intensity (≥ 50 *S. haematobium* eggs/10 ml) [17].

A participant was considered to be microhematuria-positive if blood in urine was detected by reagent strips, with the microhematuria graded as trace, + , ++ or +++ based on the color chart provided by the manufacturer of the strips. *Schistosoma haematobium* prevalence was calculated overall as well as at both the individual school and community levels. An IU was stratified as a hotspot IU if the *S. haematobium* prevalence in the school was ≥ 3.0% and/or ≥ 2.0% in the corresponding community. An IU was considered as a low-prevalence IU if the *S. haematobium* prevalence in the school was < 3.0% and < 2.0% in the corresponding community.

To predict the spatial *S. haematobium* prevalence, the study area was divided into 5350 equally sized grid points, and the prevalence per 1 km radius around the center of each grid point was determined by dividing the number of *S. haematobium*-positive individuals by the total number of community members surveyed in the 1-km radius around the center of the grid point. All HWCSs, both those with and without detected *B. globosus*, were plotted on a map indicating the predicted *S. haematobium* prevalence per 1-km radius around the center of each grid point.

To estimate the odds ratios (ORs) for a *S. haematobium* infection, generalized estimating equation (GEE) models with exchangeable correlation structure were run. Separate models were applied for the school-based survey and the community-based survey. Independent variables included in the models for both surveys were sex, age groups and distance from the school or residential house, respectively, to the next HWCS with *B. globosus*. In addition, in the model for the school-based survey, travel and reported treatment in the past 6 months were included as independent variables. In the model for the community-based survey, distance from the residential house to the next health facility was included as an additional independent variable. In the models, 95% confidence intervals (CI), and GEE with robust standard errors to account for clustering were used. Stratified by the school-based survey and community-based survey, either the schools or the communities were included in the models as clusters.

To determine the treatment coverage of the MDA conducted in August 2020 and participants’ intake of praziquantel, assessments were made of how many participants reported to have received and taken treatment in the school-based or community-based MDA, respectively. Moreover, the survey also assessed how many participants had received praziquantel at least once in their lifetime and if they considered MDA with praziquantel as a good or “not-good” intervention to fight schistosomiasis. The narrative responses were translated into English and grouped into thematic categories.

## Results

### Implementation Unit characteristics survey

The she has of the 20 study IUs reported population sizes ranging from 1030 to 7000 inhabitants, with a median of 4393. The number of housing structures reported ranged from 513 to 1400, with a median of 782. The number of public water taps per 1000 IU inhabitants reported by the she has was between zero and 74, with a median of six.

**Fig. 1:**
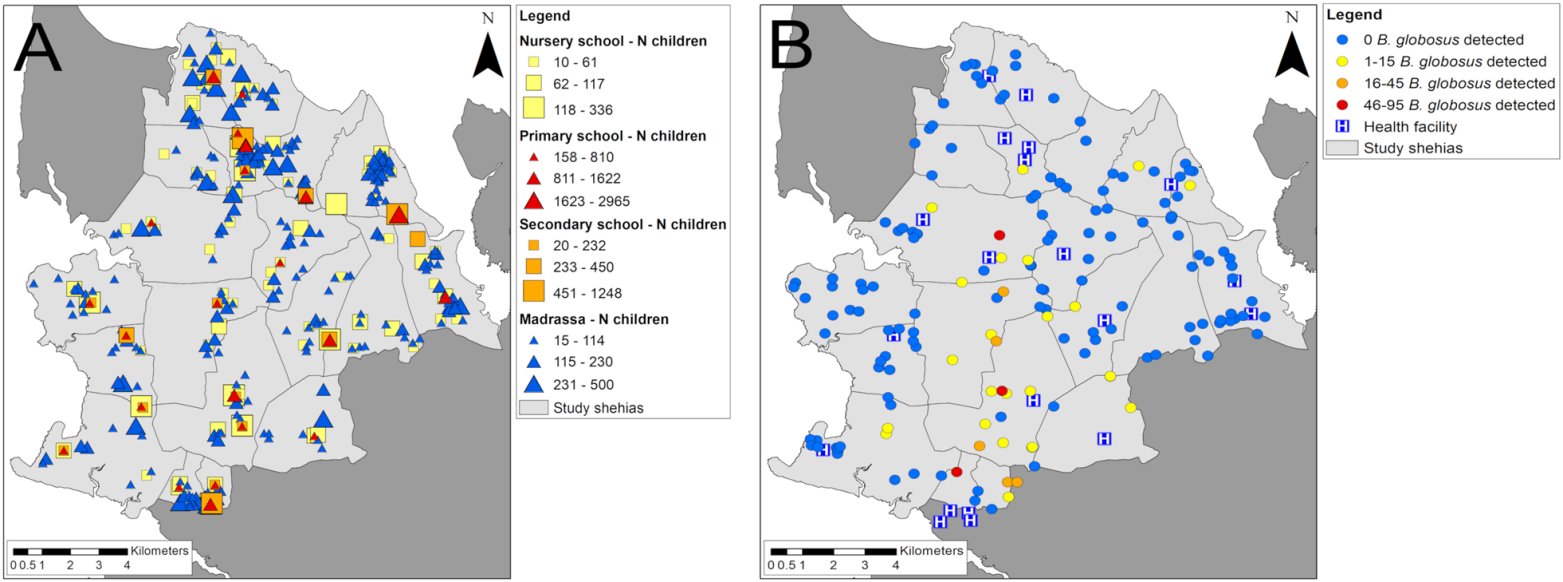
Schools, human water contact sites (HWCSs) and health facilities in the study area in Pemba, Tanzania.** A** The type and geolocations of schools with the number of children enrolled,** B** the geolocations of HWCSs with the number of *Bulinus globosus* detected and the geolocations of health facilities within the SchistoBreak study area in the north of Pemba, Tanzania. The image base map (United Republic of Tanzania—subnational administrative boundaries) was downloaded from the United Nations (UN) Office for the Coordination of Humanitarian Affairs (OCHA) services (https://data.humdata.org/dataset/tanzania-administrative-boundaries-level-1-to-3-regions-districts-and-wards-with-2012-population). The data source of the image base map is Tanzania National Bureau of Statistics/UN OCHA ROSA. The data of the image base map are published under the following license: Creative Commons Attribution for Intergovernmental Organisations (CC BY-IGO; (https://creativecommons.org/licenses/by/3.0/igo/legal code)). Written permission was obtained to use and adapt the data from OCHA. Additional shape files for the map (shehia boundaries) were provided by the Zanzibar Commission for Lands to the Zanzibar Neglected Diseases Program

A total of 367 schools were visited in the 20 IUs, of which 23 (6.3%) were primary schools, with 21 (91.3%) being public schools and two(8.7%) being private schools; 16 (4.4%) were secondary schools, with 14 (88.0%) being public schools and two (12.5%) being private schools; 89 (24.3%) were nursery schools, with 86 (96.6%) being public schools and three (3.4%) being private schools; and 239 (64.9%) were Madrassas. The location and number of enrolled students at each school is indicated in Fig. 1A.

A total of 167 HWCSs were identified in the 20 study IUs, including 112 (67.1%) located at water bodies characterized as rivers or streams and 55 (32.9%) located at lakes or ponds.* Bulinus globosus* was found in 33 (19.8%) of the 167 HWCSs. The location of HWCSs and the number of *B. globosus* collected is indicated in Fig. 1B.

At 47 (28.1%) of the 167 identified HWCSs, people were present and willing to share their opinion on the needs of the community for better protection against *S. haematobium* infections. The most highlighted need was to “*apply medicine to the water*” for reducing the amount of snails or simply to “*kill the snails*”, mentioned by people at 29 (61.7%) of the 47 HWCSs.

In the 20 study IUs, there were 21 health facilities, including 14 (66.7%) and five (23.8%) public and private PHCUs, respectively, and two (9.5%) public hospitals (Fig. 1B).

### Cross-sectional parasitological survey

#### Study participation

**Fig. 2:**
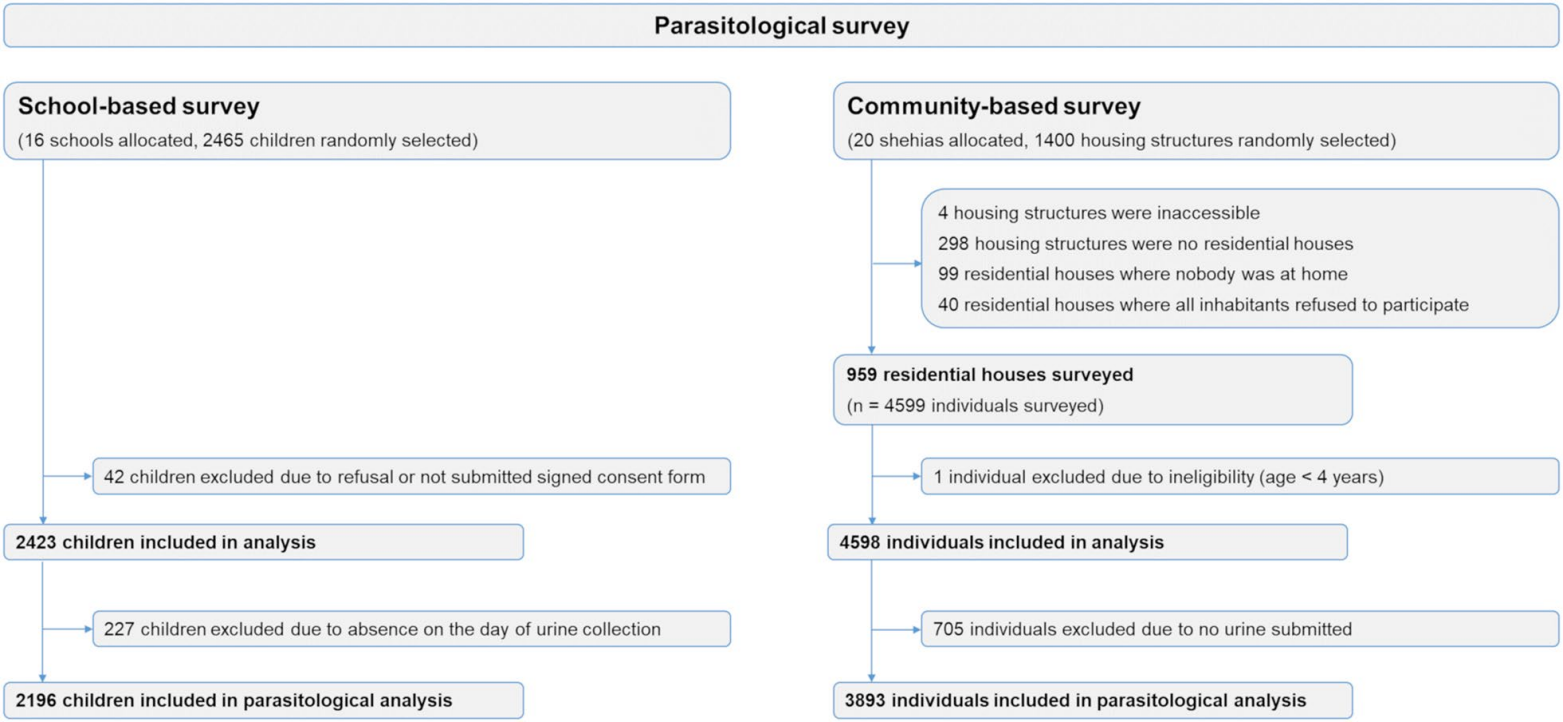
Flow diagram of individuals participating in the cross-sectional baseline survey of the SchistoBreak study in Pemba, Tanzania

In the school-based survey, a total of 2465 children from 16 schools were randomly selected and invited to participate (Fig. 2). Among these, 227 (9.4%) were absent on the day of urine collection, and 42 (1.7%) refused to participate or did not provide a signed consent form. In total, urine samples were collected and analyzed from 2196 children, of whom 1167 (53.1%) were female and 1029 (46.9%) were male (median age: 10 years, range 4–17 years).

In the community-based survey, 1400 housing structures were randomly selected (Fig. 2). Of these, 441 (31.5%) were not sampled because the structures were located in protected military areas with prohibited access, were not residential houses, had no household members at home during the time of survey or all household members refused to participate. Ultimately, 959 residential houses with a total of 4599 household members were included in the study. Among the 4599 household members, 705 (15.3%) were excluded from the analyses because of ineligibility due to their age (< 4 years old) or missing urine samples. Hence, 3893 individuals were included in the final community-based analysis, of whom 2067 (53.0%) were female and 1826 (47.0%) were male (median age: 18 years, (range: 4–87 years)) .

#### Schistosoma haematobium infection and microhematuria in schools

**Fig. 3:**
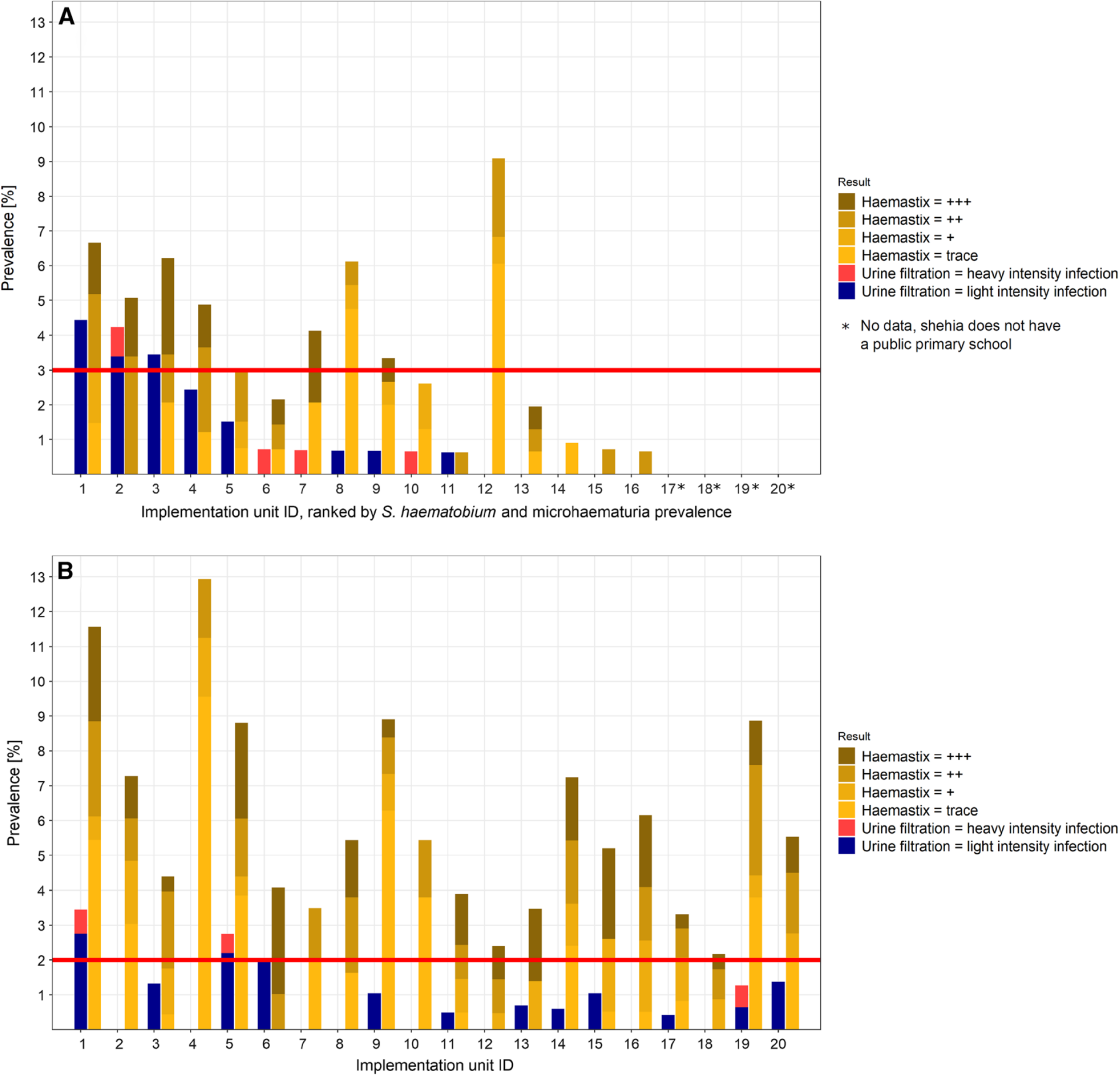
Baseline *Schistosoma haematobium* and microhematuria prevalence in the implementation units (IUs) of the SchistoBreak study in Pemba, Tanzania.** A**,** B** *Schistosoma haematobium* and microhematuria prevalence stratified by schools (**A**) and communities (**B**) in the 20 IUs of the study area in the north of Pemba, Tanzania. The ID numbers of the schools and communities correspond to the same IU. Asterisks denote those IUs (ID numbers 17, 18, 19, 20) with no main primary school to be included in the survey. Microhematuria was graded as trace, + , ++ or +++ based on the color chart provided by the manufacturer of the haemastix test strips. The red line indicates the threshold for the stratification of the IUs into hotspot or low-prevalence IUs based on urine filtration results

Among the 2196 children included in the analyses of the school-based survey, 26 (1.2%) children tested positive for *S. haematobium* by urine filtration, with heavy-intensity infections in four (0.2%) students (median egg count: 15, range 2–94). The median age of *S. haematobium*-positive children was 11 (range: 4–15) years. Among the 1167 female and 1029 male participants, 12 (1.0%) and 14 (1.4%), respectively, tested positive for *S. haematobium*. In three of the 16 surveyed schools, the *S. haematobium* prevalence was higher than the pre-set 3.0% threshold for classifying IUs as hotspots (Fig. 3A). Among the 2196 children participating in the school-based survey, 78 (3.6%) were microhematuria-positive with a median age of 11 (range: 4–15) years. Among the 1167 girls and 1029 boys, 43 (3.7%) and 35 (3.4%) tested positive for microhematuria, respectively.

#### Schistosoma haematobium infection and microhematuria in communities

Among the 3893 individuals participating in the community-based survey, 31 (0.8%) individuals tested positive for *S. haematobium*, with a heavy-intensity infection occurring in three (0.1%) individuals (median egg count of six, range 1–168). The median age of *S. haematobium*-positive participants was 10 years (range 6–52). Among the 2067 female participants, 15 (0.7%) tested *S. haematobium-*positive and among the 1826 male participants, 16 (0.9%) tested *S. haematobium*-positive. In three of the 20 IU communities, the *S. haematobium* prevalence was higher than the pre-set 2.0% threshold for classifying IUs as hotspots (Fig. 3B). Among the 3893 individuals participating in the community-based survey, 225 (5.8%) were microhematuria-positive with a median age of 20 years (rage 4–85). Among the 2067 female and 1826 male participants, 154 (7.5%) and 71 (3.9%) were microhematuria-positive, respectively.

#### Stratification in hotspot and low-prevalence implementation units

**Fig. 4:**
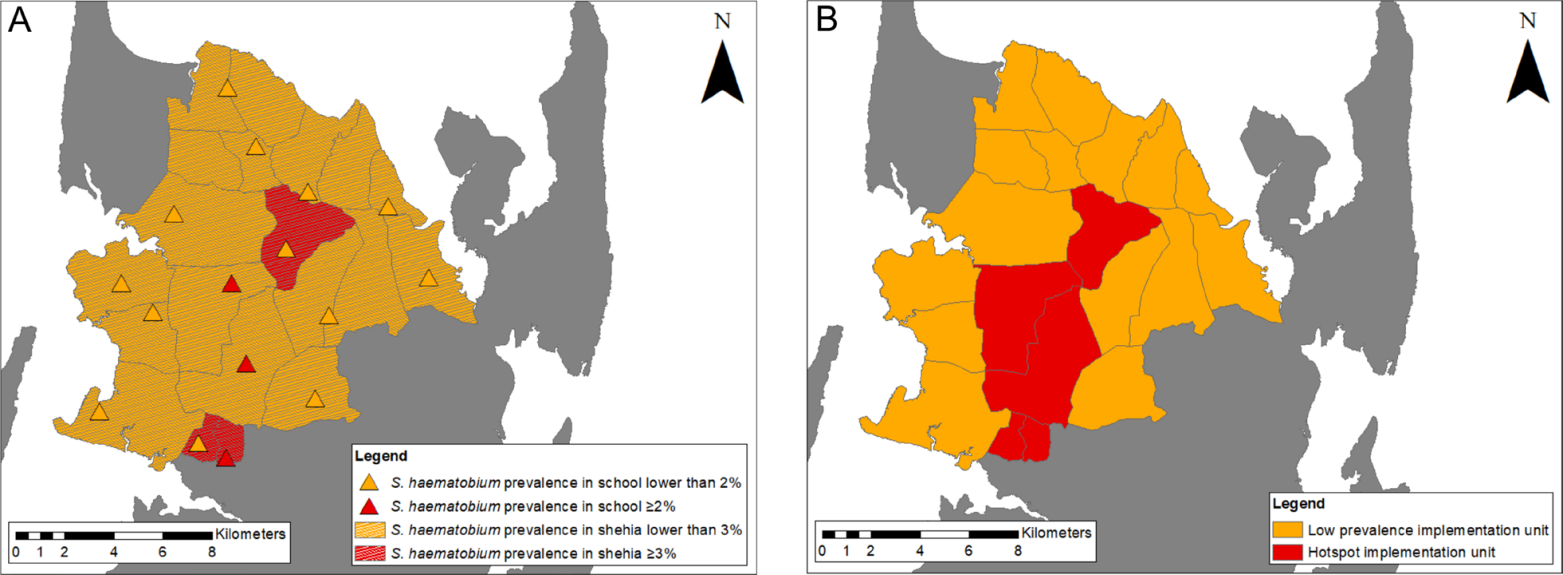
*Schistosoma haematobium* prevalence in the study sites and their classification into hotspot and low-prevalence implementation units (IUs). **A** *Schistosoma haematobium* prevalence in study schools and communities as identified during the cross-sectional parasitological survey from November 2020 to March 2021 below or above pre-defined thresholds.** B** Classification of the IUs into hotspot and low-prevalence IUs based on data shown in** A**. The image base map (United Republic of Tanzania—subnational administrative boundaries) was downloaded from the UN Office for the Coordination of Humanitarian Affairs (OCHA) services (https://data.humdata.org/dataset/tanzania-administrative-boundaries-level-1-to-3-regions-districts-and-wards-with-2012-population). The data source is: Tanzania National Bureau of Statistics/UN OCHA ROSA. The data are published under the following license: Creative Commons Attribution for Intergovernmental Organisations (CC BY-IGO; https://creativecommons.org/licenses/by/3.0/igo/legal code). Written permission was received to use and adapt the data from OCHA. Additional shape files for the map (shehia boundaries) were provided by the Zanzibar Commission for Lands to the Zanzibar Neglected Diseases Program

As indicated in Fig. 3A, B and Fig. 4A, three schools and three communities, in a total of five IUs, crossed the ≥ 3.0% and/or ≥ 2.0% prevalence threshold for *S. haematobium* infections, respectively. Our baseline survey therefore defined a total of five hotspot IUs and 15 low-prevalence IUs, respectively, for the intervention period following the survey in 2021 (Fig. 4B).

#### Geo-spatial distribution of S. haematobium infection and B. globosus in the study area

**Fig. 5:**
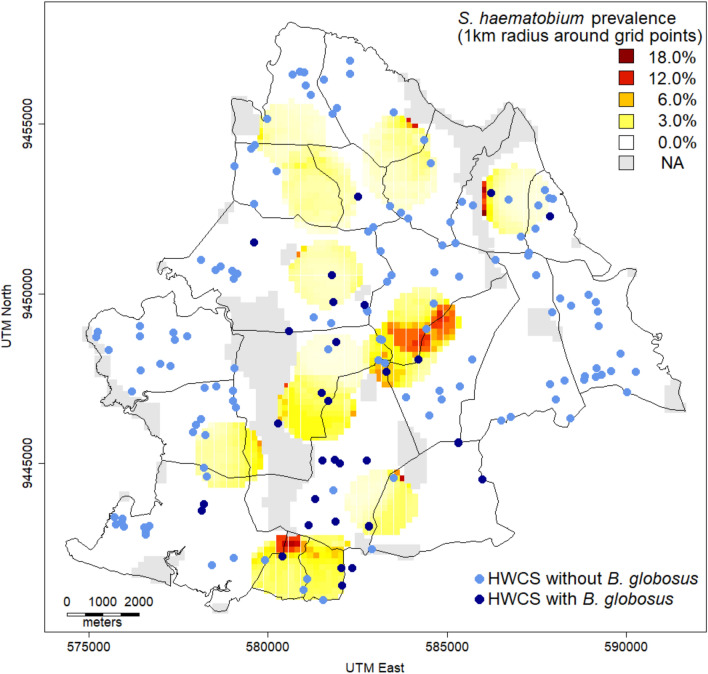
Geo-spatial distribution of *Schistosoma. haematobium* prevalence in the community-based survey and Human water contact sites (HWCSs) with and without *Bulinus globosus*. The figure shows the *S. haematobium* prevalence per 1-km radius around the center of equally sized grid points observed during the community-based survey and the spatial distribution of HWCSs with and without *B. globosus* detected during the implementation unit (IU) characteristics survey of the SchistoBreak study in the north of Pemba, Tanzania. The shape files for the map (shehia boundaries) were provided by the Zanzibar Commission of Lands to the Zanzibar Neglected Diseases Program

A geo-spatial analysis of the *S. haematobium* prevalence in the community-based survey per 1-km radius around the center of equally distributed grid points revealed that 64.8% of the geographical study area had a predicted *S. haematobium* prevalence of 0.0% (Fig. 5). In another 24.4% of the geographical study area, the predicted *S. haematobium* prevalence was  > 0.0% and  < 10.0%. In 0.5% of the geographical study area, the predicted *S. haematobium* prevalence was  ≥ 10.0%.  No *S. haematobium* data were available for the remaining 10.3% of the study area as it was uninhabited land.

The highest *S. haematobium* prevalence observed within a 1-km radius around one of the grid points was 18.2%. The median distance between a grid point plus a 1-km radius with a *S. haematobium* prevalence ≥ 1.0% and a HWCSs with *B. globosus* was 877 m. The median distance between a grid point plus a 1-km radius with a *S. haematobium* prevalence < 1.0% and HWCSs with *B. globosus* was 1616 m.

#### Risk factors for S. haematobium infection in schools

**Fig. 6:**
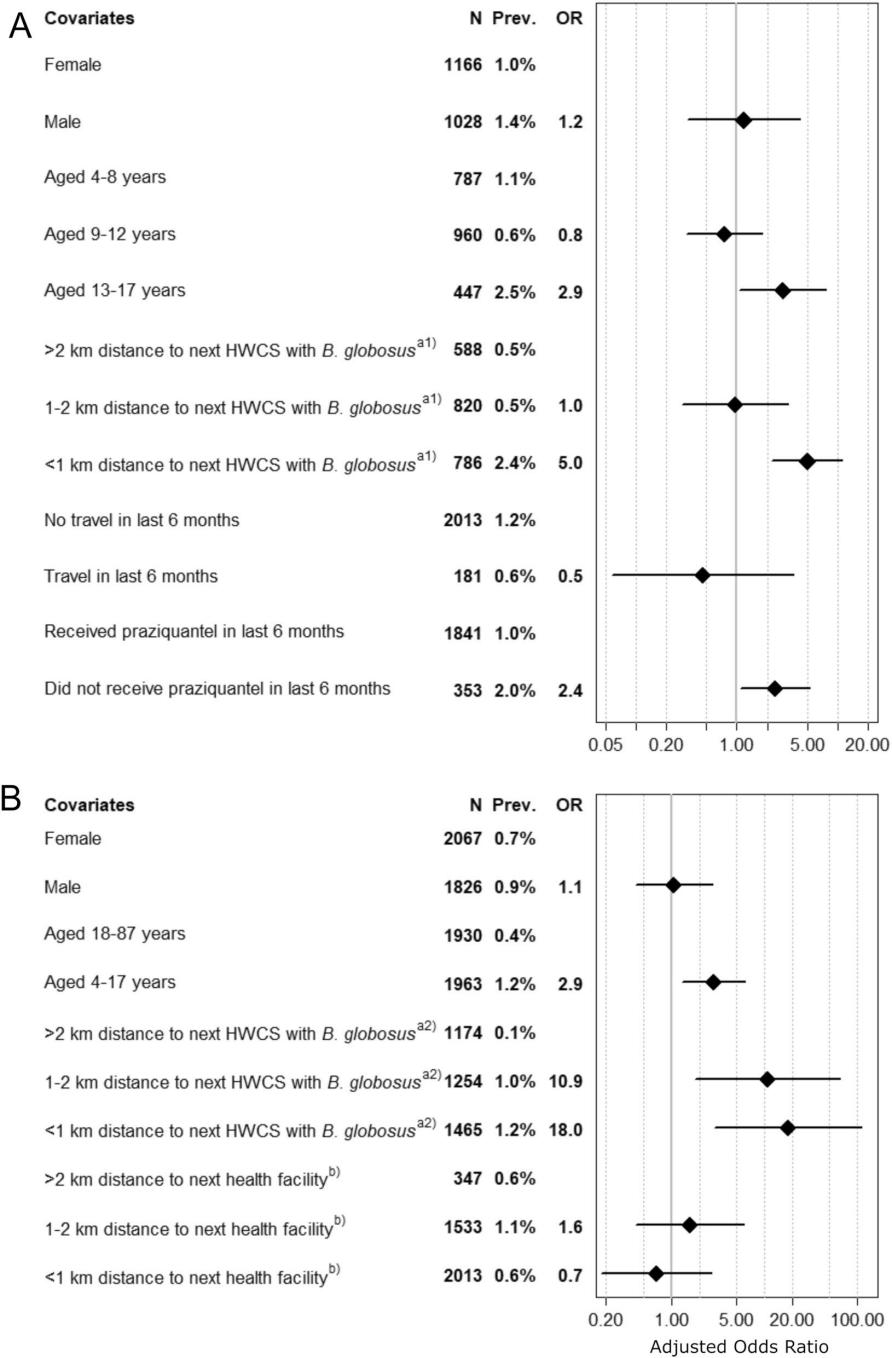
Multivariable analysis of risk factors for *Schistosoma* *haematobium* infection in participants of the SchistoBreak baseline survey.**A**,**B **The odds ratios (ORs) for a Schistosoma *haematobium* infection adjusted for various risk factors observed within the cross-sectional school-based (**A**) and community-based (**B**) surveys conducted from November 2020 to March 2021 in the north of Pemba, Tanzania. a1) indicates the distance from the school to the nearest human water contact site (HWCS) where *Bulinus globosus* was detected during the implementation unit (IU) characteristics survey. a2) indicates the distance from the residential house of the individual to the nearest human water contact sites (HWCS)  HWCS where *B. globosus* was detected during the IU characteristics survey. b) indicates the distance from the residential house of the individual to the health facility. Abbreviations: Prev., prevalence

In the school-based survey, a total of 2194 participants were included in the GEE model analyses. No statistically significant association was revealed between* Schistosoma haematobium* infection and sex (Fig. 6A). Children aged 13–17 years had a significantly higher chance (OR: 2.9; CI: 1.1–7.6; prevalence: 2.5% *versus* 1.1%) of acquiring a *S. haematobium* infection than children aged 4–8 years. Children aged 9–12 years did not have a higher chance of being infected with *S. haematobium* than children aged 4–8 years. Moreover, children enrolled in a school located < 1 km away from the closest HWCS where *B. globosus* was detected had significantly higher odds (OR: 5.0; 95% CI: 2.3–11.1; prevalence: 2.4% vs 0.5%) of being infected with *S. haematobium* than children enrolled in a school that was located > 2 km away from the closest HWCS with *B. globosus*. The OR for children enrolled in a school 1–2 km away from the closest HWCS where *B. globosus* was detected was 1.0 (95% CI: 0.3–3.2; prevalence: 0.5%) compared with children enrolled in a school > 2 km away from the closest HWCS with *B. globosus*. No statistically significant association was determined for travel in the last 6 months. Children who had not received praziquantel during the previous 6 months had 2.4-fold higher odds (95% CI: 1.1–5.3; prevalence: 2.0% vs 1.0%) of being infected with *S. haematobium* than children who had received treatment in the previous 6 months.

#### Risk factors for *S. haematobium* infection in communities

In the community-based survey, a total of 3893 individuals were included in the GEE model analyses. No statistically significant association was determined between *S. haematobium* infection and sex (Fig. 6B). Children (4–17 years) had significantly higher odds of a *S. haematobium* infection (OR: 2.9; 95% CI: 1.3–6.2; prevalence: 1.2% vs 0.4%) than adults (aged ≥ 18 years). Individuals residing 1–2 km from a HWCS with *B. globosus* had a significantly higher chance of a *S. haematobium* infection (OR: 10.9; 95% CI: 1.8–64.4; prevalence: 1.0% vs 0.1%) than individuals living > 2 km away from the next HWCS with *B. globosus*. The odds for a *S. haematobium* infection were highest in individuals who resided < 1 km away from a HWCS with *B. globosus* (OR: 18.0; 95% CI: 2.9–111.0; prevalence: 1.2% vs 0.1%) compared with individuals living > 2 km away from a HWCS with *B. globosus*. No statistically significant association was observed between a *S. haematobium* infection and the distance of residential houses to the next health facility.

#### MDA coverage and perception of MDA as a good or not-good intervention

In the school-based survey, 2015/2423 (83.2%) children reported that they were treated with praziquantel during the last school-based MDA in August 2020.

**Fig. 7:**
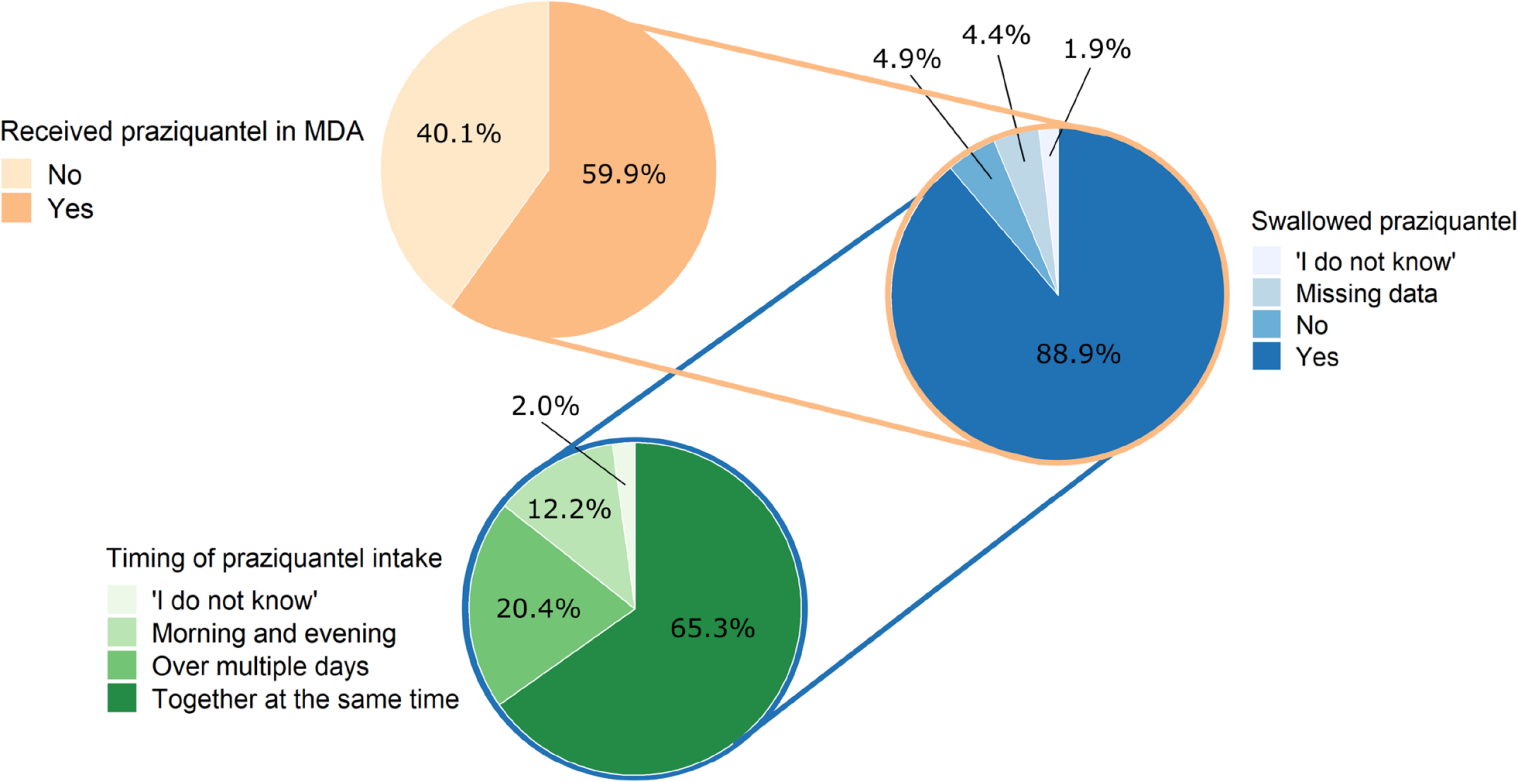
Coverage and praziquantel intake among adults during the community-based mass drug administration (MDA) in August 2020

In the community-based survey, 574/958 (59.9%) individuals reported that they had received praziquantel during the last community-based MDA in August 2020 (Fig. 7). Among those 574 individuals who reported to have received the drug, 510 (88.9%) claimed to have swallowed the received tablets, with 333 (65.3%) individuals reporting that they took all of the tablets at the same time, 104 (20.4%) reporting that they took the tablets over multiple days, 62 (12.2%) reporting that they split the tablet intake to the morning and evening of the same day and 11 (2.2%) not answering the question. Among the 64 individuals who did not swallow the tablets, the two most common reasons reported for not swallowing praziquantel were being pregnant (13/64; 20.3%) and feeling healthy (5/64; 7.8%). Among all 958 adult participants interviewed, 333 (34.8%) received praziquantel and took all tablets at the same time, in line with WHO recommendations [4].

Moreover, 786/958 (82.0%) interviewed participants reported that they had received praziquantel at some time in their life. Among those who had ever received praziquantel in their life, 548 (69.7%) perceived MDA as a good intervention, 87 (11.1%) perceived MDA as not a good intervention, 143 (18.2%) were indecisive and eight (1.0%) did not answer. The most common thematic reasons for considering MDA a good intervention were “*It is good for the health*” (327/501; 65.3%) and ”*One saves time/money/stress of going to the hospital to get the drugs as the drugs are delivered at home*” (74/501; 14.8%). The major reasons why MDA was not perceived as good were “*One is not tested for schistosomiasis before receiving the treatment*” (56/78; 71.8%) and “*The drug distributors are no medical personnel*” (6/78; 7.7%).

## Discussion

Schistosomiasis elimination has been given a high priority in the WHO guidance documents published in 2020 and 2022 [1, 4]. Schistosomiasis as a public health problem has been eliminated on Pemba Island since 2017 [5, 6]. As is part of the Zanzibar archipelago, United Republic of Tanzania, Pemba is included on the WHO list of countries that have been targeted for interruption of schistosomiasis transmission by 2030. The SchistoBreak project implemented in the north of Pemba between 2020 and 2024 aims to assess new tools and strategies for moving from elimination as a public health problem towards interruption of transmission [8, 16].

The results of the baseline survey confirmed a low overall *S. haematobium* prevalence and percentage of heavy infection intensities, respectively, in both schools (1.2% and 0.2%) and communities (0.8% and 0.1%) of the SchistoBreak study area. The community-based survey revealed that in most (89.2%) of the geographical study area, the prevalence was <10.0% and that, according to the new WHO guidelines, the area can be considered to be a low-prevalence setting [4].

There is a large number of freshwater bodies in the study area, and many of these are infested with the intermediate host snail *B. globosus*. As such, these freshwater bodies are a risk for transmission and a challenge for elimination [18, 19]. The GEE model confirmed that children attending a school or community members living in a house within a 1-km radius of a HWCS with *B. globosus* had a 5- or 18-fold higher chance of being infected with *S. haematobium*, respectively. Our results are in line with those of other studies conducted in Zanzibar and Nigeria that also observed a positive relationship between *S. haematobium* infection and decreasing distance to HWCSs with *B. globosus* or freshwater bodies in general [3, 20–22].

These findings can have considerable implications for cost-effective schistosomiasis risk assessment and micro-targeting interventions in close-to-elimination settings: to identify potential high-transmission of *S. haematobium* sites*,* rigorous and repeated snail surveys at HWCSs should be conducted. Then, if *B. globosus* is detected, focal snail control measures could be applied coupled with focal MDA or test-and-treat targeting individuals living or attending schools within a 2-km radius around these HWCSs. Snail control measures involving niclosamide is recommended by WHO as a cost-effective intervention tool that has a large impact on *S. haematobium* infections [4, 23–25]. Many individuals (61.7%) interviewed at the HWCSs during our IU characteristics survey proposed applying “medicine,” i.e. niclosamide, to the HWCSs to target the intermediate host snails*,* which indicates a high level of community acceptance for snail control in the study area. Focal snail control has been implemented successfully in other settings, such as Egypt and Mali [26, 27]. In similar low-prevalence settings for malaria, focal MDA plus vector control have been applied to reduce transmission [28, 29] and reactive case detection, and focal MDA against malaria were shown to have a high community acceptance in Namibia [28, 29].

There is a long history of large-scale MDA in Pemba and in other countries as preventive chemotherapy is recommended by WHO as an intervention to control morbidity of schistosomiasis and to advance towards the elimination of this disease as a public health problem in communities with a *Schistosoma* prevalence ≥ 10% [4, 10, 30]. The continuation of regular preventive chemotherapy is also recommended among existing control programs as they move towards interruption of transmission, such as in Pemba [4]. While the self-reported coverage and compliance with directly observed praziquantel intake in the school-based MDA in 2020 was 83.8% among schoolchildren in our study in Pemba, treatment coverage among adults in the community-based door-to-door approach was only 59.9%. Ultimately, only 34.8% of the targeted adult population received and swallowed all of the tablets at the same time—and hence as a single oral dose—in line with WHO recommendations [4], which is worryingly low and might point to treatment fatigue. The main reasons for not swallowing any of the received praziquantel tablets were pregnancy and feeling healthy at the time. All adult participants who ever participated in an MDA in the past were asked about their opinion about MDA. Among those who considered MDA not as a good intervention, their qualitative response was “*MDA is not a good intervention because one is not tested for schistosomiasis before receiving the treatment.*” Hence, offering praziquantel in health facilities, including mother and child health care units, by medical personnel [31] and using a test-and-treat strategy instead of preventive chemotherapy without prior diagnosis might increase compliance among adults. However, to date, no sensitive and specific test for *S. haematobium* detection at the point-of-care exists, presenting a considerable challenge for low-prevalence settings where most people are only slightly infected [32–36]. The development of a highly sensitive and specific test that is affordable and simple and can also be used in health facilities in low-prevalence areas is an urgent need when moving towards interruption of transmission and post-elimination surveillance [37–40].

In the SchistoBreak study, we will explore some of the intervention approaches recommended by WHO in their new guidelines and/or those suggested above. We are confident that our study will reveal important insights into and provide evidence for their feasibility and contribution to the goal of interrupting transmission. While the baseline survey already has produced valuable results, there are a number of limitations worth highlighting. First, the snail surveys during the IU characteristics survey were conducted only once per HWCS. Hence, there may be HWCSs where *B. globosus* was not detected during the time of visit but which are a transient habitat for the snails, possibly distorting our results. Second, *S. haematobium* prevalence was determined by a single urine filtration per person. The low sensitivity of the test to detect light-intensity infections could result in an underestimation of the true prevalence [36]. Third, in the school-based survey, the GEE model was based on the distance of the children’s school to HWCSs with *B. globosus* and not the children’s homes, which might have provided more accurate results than the distance from the school.

## Conclusions

We found that the overall prevalence of *S. haematobium* in the north of Pemba is low, but the many HWCSs with *B. globosus* situated close to schools or houses represent a high risk for rebounding transmission and recrudescence of infection. Furthermore, while treatment coverage among schoolchildren was high, the correct intake of praziquantel during MDA was low among adults. To maintain the gains made and accelerate towards interruption of *S. haematobium* transmission in Pemba and elsewhere, there is a need for new processes and integrated intervention strategies that are cost-effective and accepted in the community, such as intensified  snail surveys, focal snail control, focal MDA and/or targeted test-and-treat approaches.

## Supplementary Information

**Additional file 1:** Additional data that support the findings of schools within the IU characteristics survey from February 2020 until July 2020 (CSV).

**Additional file 2:** Additional data dictionary that explains the data of schools collected during the IU characteristics survey from February 2020 until July 2020 (TXT).

**Additional file 3:** Additional data that support the findings of water bodies within the IU characteristics survey from February 2020 until July 2020 (CSV).

**Additional file 4:** Additional data dictionary that explains the data of water bodies collected during the IU characteristics survey from February 2020 until July 2020 (TXT).

**Additional file 5:** Additional data that support the findings of implementation units within the IU characteristics survey from February 2020 until July 2020 (CSV).

**Additional file 6:** Additional data dictionary that explains the data of implementation units collected during the IU characteristics survey from February 2020 until July 2020 (TXT).

**Additional file 7:** Additional data that support the findings of health facilities within the IU characteristics survey in September 2021 (CSV).

**Additional file 8:** Additional data that explains the data of health facilities collected during the IU characteristics survey in September 2021 (TXT).

**Additional file 9:** Additional data that support the findings of the baseline survey from November 2020 until February 2021 (CSV).

**Additional file 10:** Additional data dictionary that explains the data collected during the baseline survey from November 2020 until February 2021 (TXT).

## Data Availability

All data generated or analysed during this study are included in this published article and its Additional information files (Additional file 1, 2, 3, 4, 5, 6, 7, 8, 9, 10).

## Abbreviations

CI: Confidence interval
GEE: Generalized estimating equation
HWCS: Human water contact site
IU: Implementation unit
MDA: Mass drug administration
ODK: Open data kit
OR: Odds ratio
PHCU: Primary health care unit
